# Deliberations About Genomic Research and Biobanks With Citizens of the Chickasaw Nation

**DOI:** 10.3389/fgene.2020.00466

**Published:** 2020-05-14

**Authors:** Justin Reedy, Jessica W. Blanchard, Justin Lund, Paul G. Spicer, Christie Byars, Michael Peercy, Bobby Saunkeah, Erika Blacksher

**Affiliations:** ^1^Department of Communication and Center for Risk & Crisis Management, University of Oklahoma, Norman, OK, United States; ^2^Department of Anthropology, University of Oklahoma, Norman, OK, United States; ^3^Chickasaw Nation Department of Health, Ada, OK, United States; ^4^Department of Bioethics & Humanities, University of Washington, Seattle, WA, United States

**Keywords:** genomics, biobanks, indigenous peoples, community-based participatory research, deliberation

## Abstract

Amid the rapid growth of precision medicine and biobanking initiatives, there have been few efforts at cataloging the implications of these initiatives for Indigenous communities. A consortium involving a university and three American Indian/Alaska Native (AIAN) community partners is working to promote deliberation and dialog in AIAN communities about the potential benefits and risks of genomic research for those communities. The first of the consortium’s three planned deliberations was held in September 2018 with citizens of the Chickasaw Nation, a federally recognized tribe in south-central Oklahoma with a full-service medical center and growing research capacity and oversight. Consortium members and the Chickasaw Nation Department of Health Administration designed a deliberative forum for Chickasaw citizens to consider the potential benefits and risks of participating in genomic research and biobanks. In this manuscript, we describe the deliberative method used in this event and report on the ideas discussed during the tribal citizens’ deliberations. Chickasaw citizens identified many risks and benefits associated with genomic research and biobanks, including the potential for medical advancements that might benefit the Chickasaw community as well as the possibility of discrimination against the Chickasaw people. Although participants thought the potential benefits outweighed the potential risks, that moral calculation was contingent on whether control of the research and biobanks rested with Chickasaw leadership, researchers, and citizens.

## Introduction

Amid excitement about the prospect that precision medicine research will yield targeted treatments based on individual genomic and environmental variation, concerns have been raised about the lack of racial and ethnic diversity among participants in genomic studies and biobanks (Cohn et al., 2017; Cornel and Bonham, 2017). In the absence of sufficient diversity, including American Indian and Alaska Native peoples (AIAN), insights into underlying disease biology and subsequent treatment strategies may not be relevant to minority groups, who experience disproportionate rates of disease and premature death (Popejoy and Fullerton, 2016; Claw et al., 2018).

American Indian/Alaska Native communities have concerns about precision medicine beyond its incapacity to ameliorate health disparities (Bayer and Galea, 2015; West et al., 2017). Ethical lapses in research with tribal people are manifold and serious, including the perpetuation of the objectification of AIAN peoples (Bowekaty and Davis, 2003; Dalton, 2004; Burhansstipanov et al., 2005; Strickland, 2006; Drabiak-Syed, 2010; Mello and Wolf, 2010; Boyer et al., 2011; Christopher et al., 2011; Harding et al., 2012; Kelley et al., 2013; Morton et al., 2013; Claw et al., 2018). Even still, genomic research remains of interest to some Native communities. The future of genomics in Indian Country will need to be founded on a dedication to respect, collaboration, and dialog between researchers and tribal partners with a goal of building tribal capacity and there exist promising examples (Reardon, 2017; Claw et al., 2018; Dirks et al., 2019).

Among efforts to cultivate dialog about the potential benefits and risks of genomic research for AIAN communities is the Center for the Ethics of Indigenous Genomic Research (CEIGR) consortium, a National Institutes of Health Center of Excellence in ELSI Research (CEER) based at the University of Oklahoma and in collaboration with the Chickasaw Nation in Oklahoma, Southcentral Foundation in Alaska, and Missouri Breaks Industries Research Incorporated in South Dakota. We collectively agreed to design and implement public deliberations at each of these partner sites. The Chickasaw Nation deliberation was the first, taking place on September 14–15, 2018, with enrolled members of a federally recognized tribal nation residing within the Chickasaw Nation boundaries, a jurisdiction that spans a 13-county region in south-central Oklahoma. The Chickasaw Nation has a full-service medical center, a Division of Research and Public Health, and one of three federally registered tribal Institutional Review Boards in the state. The potential of genomics research to empower political interests economically, medically, and academically has been recognized and is currently being explored by communities around the world (Tarkkala and Tupasela, 2018). In the context of growing research capacity and oversight, the CEIGR deliberation team and Chickasaw Nation Department of Health Administration designed a deliberative forum for Chickasaw citizens to consider the potential benefits and risks of participating in genomic research and biobanks. Chickasaw Nation collaborators also sought to assess the utility of public deliberation as an approach to engaging their communities on issues about their health care system and future research directions. The resulting deliberation provided insight into Chickasaw Nation citizen views on potential benefits and risks of genetic research and the potential utility of deliberative engagement for the Chickasaw Nation.

## Method

Following approval from the Chickasaw Nation IRB, health system administration, and tribal public affairs, we set out to conduct the Chickasaw Nation forum, and designed the event to adhere to key tenets of public deliberation. Although there are varied definitions of public deliberation, this approach typically convenes diverse people from varied backgrounds to learn, discuss, and carefully weigh multiple perspectives about an issue that affects them and offer reasons for their positions and priorities (Burkhalter et al., 2002; Chambers, 2003). Public deliberation has recently gained traction in the US health sector and bioethics community as policy makers have sought informed input from stakeholders on numerous topics, including genomic research (O’Doherty et al., 2012; Abelson et al., 2013; Dry et al., 2017). In particular, deliberation has proven useful for letting citizens weigh in on plans for prospective biobanks and how those facilities should be governed and regulated (O’Doherty and Burgess, 2009; Longstaff and Burgess, 2010; O’Doherty et al., 2012). Deliberations often yield informed and egalitarian discussions, and they are particularly valued by members of minority groups (Goold et al., 2005; Gastil et al., 2010; Knobloch et al., 2013; Wang et al., 2015), but there is little work examining public deliberation in indigenous contexts (Carson et al., 2013).

**FIGURE 1:**
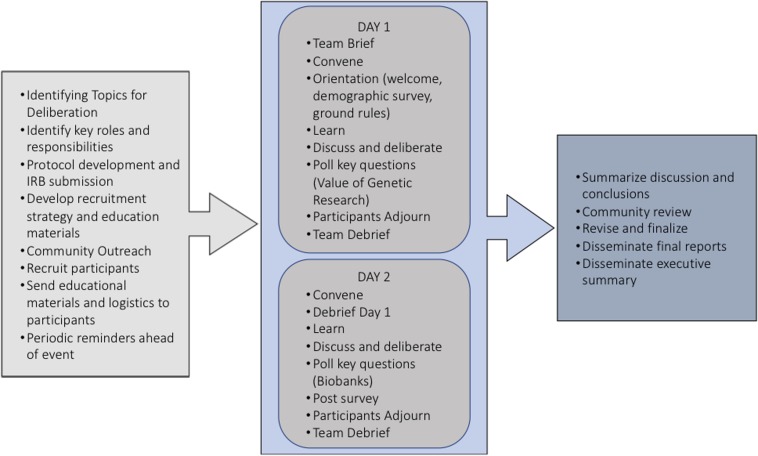
Schematic for deliberation process.

Deliberants met for a total of 10 h over 2 consecutive days. Participants explored potential risks and benefits of participation in genomic research on day 1, followed by a discussion of potential risks and benefits of tribal participation in biobanks (Figure 1). The focus and size of discussions were varied by using plenary discussion and small group discussion. Plenary sessions included brief informational presentations, presented by an Epidemiologist and tribal IRB Administrator, to provide background information and answer factual questions on topics related to tribal research protections and biobanks. The expert presentations were designed in concert with the trained deliberative facilitator (who is a bioethicist and not affiliated with Chickasaw Nation) to ensure sufficient neutrality as to not unduly influence the deliberants. Small group sessions were grounded in hypothetical scenarios designed to introduce real world concerns and issues related to the deliberation topics. The deliberation team and the tribal partners planned carefully to ensure that deliberants were presented with fact-based information and any efforts to ask value-based questions or judgements of the presenter were promptly diverted back to the deliberants to discuss. These mixed formats helped generate different group dynamics, cross-pollination of ideas, and speaking opportunities for reticent deliberants. The ideas generated by deliberants were recorded on flip charts throughout the plenary sessions and deliberant checking was used to ensure accuracy. In addition, two observers from the deliberation team (who were not affiliated with Chickasaw Nation) were present and took field notes on the process. The results of key considerations presented below are based in part on flip chart notes, the analysis of those field notes. These represent what O’Doherty and Burgess (2009) call “deliberative output” from the forum rather than a systematic qualitative analysis of deliberants’ discussions, which will be reported elsewhere and are beyond the scope of the current manuscript.

## Results

### Deliberants

Sixteen tribal citizens residing in the Chickasaw Nation boundaries participated in the forum (10 women, 6 men), 15 of whom (10 women, 5 men) participated on both days; one person was unable to return the second day. Deliberants ranged in age from 25 to 74, male and female representation, and educational levels of participants ranged from high school to post-baccalaureate. All participants had some level of exposure to health-related research and all had familiarity with the Chickasaw Nation health system. Participants received compensation in the form of gift cards for their time and contributions. Tribal citizens from diverse Chickasaw communities were represented.

### Recruitment

A two-phased recruitment strategy was used to reach a diverse sample of participants at least 18 years of age, enrolled members of a federally recognized tribal nation, and residing within the Chickasaw Nation geographical boundaries. Chickasaw Nation boundaries span a 13-county jurisdiction that is home to both Chickasaw and other tribal citizens, and Chickasaw Nation health facilities serve any individual enrolled in a federally recognized tribe.

The first phase of recruitment took place within Chickasaw Nation health facilities, and included advertisements and secure email distributions circulated within the Chickasaw Nation health facilities. This broad approach to recruitment ensured that participation included tribal citizens who utilize and are familiar with Chickasaw Nation services.

The second phase of recruitment involved a more targeted effort to oversample for representation of Chickasaw Nation citizens living in diverse communities across tribal boundaries to achieve diversity of perspectives in the deliberation. Four specific communities within Chickasaw Nation boundaries were selected based on their proximity to operating tribal health clinics or the tribal hospital. Recruitment occurred at tribal community centers in each of these select locations. It was critical that on the ground recruitment was facilitated by someone familiar with the Chickasaw Nation health systems and also familiar with each community. A Chickasaw Tribal member who works as a Research Assistant within the tribal research and public health department did all recruitment for this Chickasaw Nation deliberation.

### Deliberative Outcomes and Quality of Deliberation

Deliberations culminated in polling participants on a question about the relative balance of risks and benefits of tribal involvement in genomic research (day 1) and biobanks (day 2). Participants took a post-deliberation survey to help us assess the quality of the deliberation event, which is a common technique for ensuring that a forum is of sufficient deliberative quality (Goold et al., 2005; Knobloch et al., 2013). The survey results showed that a strong majority of participants had positive views of the forum and strongly endorsed the value of community deliberations, with all 15 full participants agreeing or strongly agreeing that facilitators made sure all opinions were heard and that people respected each other’s opinions. Nearly all (14 out of 15) said they spoke as much as they wanted, and nearly all (14 out of 15) agreed that the discussion led them to change some of their opinions on the topic. All agreed that more events like this should be held as a way of getting the views of people in the community, and would recommend that friends and family participate in future activities like this. In addition, a deliberation expert served as one of the two observers of the event and found that the forum performed well on several criteria commonly used for assessment of deliberative quality, such as building a good base of information, providing equal speaking opportunity, and respectful interaction between participants (Knobloch et al., 2013).

### Do the Potential Benefits of Tribal Participation in Genomic Research Outweigh the Potential Risks (or Vice Versa)?

A majority (15 out of 16) concluded that the potential benefits outweighed the potential risks. Participants discussed a wide range of potential risks and benefits, which are reflected in the considerations they identified as relevant to weighing the potential benefits and risks of genomic research in their community (see Table 1).

**TABLE 1:** Considerations for weighing the potential risks and benefits of tribal participation in genomic research.

Data control	Who will control the data? Where will it be stored, will it be secure, and will personal privacy and identity be protected?
Data sharing	Who has access to the information? What are the research questions and do they reflect Native people’s concerns?
Benefits of research	Is the research relevant to Native people, will it have a measurable positive impact on Native health outcomes, and will Native people have access (e.g., cost, geography) to the treatments or prevention strategies that might result from the research? Will research provide more knowledge about Chickasaw health and disease (particularly high rates of diabetes) and yield better treatments and prevention strategies? Will the research provide benefits to society at large? Opportunities for Native researchers to train for and conduct this type of research.
Necessity of research	Is genetic research necessary to address the condition/disease being studied? Questions about the value and relevance of studying Native Americans.
Misuse of genetic information	Could it be used to discriminate against individuals (e.g., insurance or employment) or against Chickasaw people (stigmatization of entire group), or against humanity?
Immoral uses of genetic research	Could it be used to alter biological life (human and non-human) in ways that transgress moral boundaries or “play God”?

### Do the Potential Benefits of Tribal Participation in Biobanks Outweigh the Potential Risks (or Vice Versa)?

A majority (13 out of 15) concluded that the potential benefits outweighed the potential risks. Deliberants identified several areas of concern related to participation in biobanks, which are reflected in the considerations they identified in weighing the potential risks, and benefits of biobanks (see Table 2).

**TABLE 2:** Considerations for weighing the potential risks and benefits of tribal participation in biobanks.

Consent	How would consent for use of data be handled? Would the individual have the opportunity to be informed about and consent to each new use? Would tribal review of secondary uses provide adequate protection? Would an approach to consent other than individual or tribal consent provide adequate protections (e.g., based on type of research or disease)?
Data sharing	Who decides who has access to the information and which researchers will be given access? What are the researchers studying and is it in Native people’s best interest?
Long-term storage	Why do data need to be stored long-term? Would long-term storage increase the risk of misuse?
Privacy	Will individual privacy and confidentiality be secured and protected against misuse (e.g., discrimination in employment or health/life insurance)?
Cost/opportunity costs	How much would it cost to build, maintain, and secure a Chickasaw biobank? Would these costs distract from other worthy investments in Chickasaw people and programs?
Psychological burdens	Would genetic information about self or family cause psychological harms (e.g., distress, fear, anger, and sadness)?
Health benefits	Will research provide more knowledge about Chickasaw health and disease (particularly high rates of diabetes) and yield better treatments and prevention strategies? Will the research provide benefits to society at large?
Opportunities for advancement of Chickasaw Nation	Biobanks may provide opportunities to build infrastructure and capacity, create jobs and bring leading Chickasaw researchers back home to the region, advance ground-breaking research, and alleviate mistrust.

## Discussion

The Chickasaw Nation deliberation identified many risks and benefits associated with tribal participation in genomic research and biobanks. Many of the risks that engaged the group have been identified before by tribal communities. For example, deliberants expressed concerns about who would have access to their data and the trustworthiness of the researchers, whether the research would benefit their communities and their privacy would be protected, and how consent would be obtained. The majority of deliberants ultimately thought the potential benefits of genomic research—in particular, knowledge about Chickasaw health and disease, treatments and prevention strategies, and opportunities for native researchers to do genomics research—outweighed the risks. However, that optimism was contingent on whether control of the research and data stored in biobanks rested with Chickasaw people—tribal leadership, researchers, and citizens. Many participants’ concerns about biobanks were greatly eased by the prospect of a Chickasaw Nation biobank. However, some noted that even though tribal oversight and protection would likely protect them as individuals and as a community, it might not be sufficient to protect them in the future should the tribal government leadership change.

Other participants raised a separate concern about the opportunity costs that might accompany the creation of a tribal biobank, such as diverting funds from other worthy tribal health and social programs. This line of discussion led participants to consider an alternative approach to biobanks, namely, to provide tribal data (biological samples and other health data) to non-Chickasaw biobanks (e.g., University of Oklahoma or All of Us) but to subject these to rigorous tribal standards of protection. Deliberants did not use the plenary sessions to elaborate on the specifics of these tribal protections during this event, but the sovereign status of federally recognized tribes means that tribal entities have the authority and mechanisms (i.e., tribal Institutional Review Boards) to determine the standards whereby tribal data is collected, utilized, and shared. This deliberation work was designed in consultation with the Chickasaw Nation IRB, and deliberants expressed confidence and trust in the tribal IRB to review and regulate research in a culturally respectful manner.

It is important to underscore that although many deliberants valued the promise of genomic research, this view was not unanimous. Those for whom the concerns outweighed the promise were consistent throughout the deliberation. And, even those who favored tribal participation in genomic research and biobanking did so while acknowledging the dangers. Participants traced some of their concerns to a legacy of abuses inflicted on Native peoples by the US federal government and the historical trauma they experience and embody as a result. As one deliberant said: “I think that’s a big issue, going back to the historical trauma. That…probably as a whole, we don’t trust the (US federal) government, we don’t trust their decisions in a lot of things. And so…to do something that’s going to affect the whole tribe because somebody outside said this is a good thing, well, we’ve heard that before.”

The intergenerational nature of historical trauma related to biomedical exploitation and unethical research practices continues to perpetuate apprehensions about genetic research in some AIAN communities. The continued underrepresentation of AIAN peoples in genome-wide association studies also means that these communities are less likely to benefit from any potential utilities from such research (Popejoy and Fullerton, 2016; Claw et al., 2018). Still, some Indigenous communities continue to be interested in and are pursuing genetic research in spite of persistent concerns (Claw et al., 2018). In an era of more open science and population-wide precision medicine research (e.g., All of Us), its pursuit should be done in close collaboration and dialog with tribal leadership and communities and in accordance with tribal values and guidelines (Reardon, 2017; Claw et al., 2018; Dirks et al., 2019).

Deliberation has been used to promote the inclusion of diverse interests in public forums related to biobanks (O’Doherty and Burgess, 2009). The deliberative effort described here is particularly unique in that it was designed and conducted in collaboration with a tribal partner, for the purpose of eliciting only tribal perspectives. Tribal citizens maintain diverse perspectives about genetic research, and the deliberative event provided a dynamic space for participants to engage with new information and to interact with one another about the impact of genetic research, an opportunity that the deliberants valued greatly.

This study has several limitations. First, this forum was fairly modest in size (15 full participants on days 1 and 2), and though the group was demographically diverse, given the size of this forum it could not be fully representative of the Chickasaw Nation public. Future research in tribal communities in Oklahoma and elsewhere that garners views from a larger segment of the public could help improve our understanding of American Indian views on these issues. Second, the health and biomedical issues faced in Indian Country are wide-ranging, many of which have little do with genomics research. This forum did not frame AIAN health issues broadly, focusing instead on genomic research and biobanks. This framing could have led deliberants to be overly focused on these topics rather than health issues of greater importance or salience to them.

## Data Availability Statement

In order to maintain participant privacy and comply with institutional review board requirements, the datasets analyzed for this study are not publicly available.

## Ethics Statement

The studies involving human participants were reviewed and approved by The Chickasaw Nation Institutional Review Board. The patients/participants provided their written informed consent to participate in this study.

## Author Contributions

JR, JB, JL, and EB were the primary writers of the article text. PS is the primary investigator on the grant funding this research and assisted with the intellectual development of this article. JR, JB, JL, EB, MP, BS, and CB helped design this study and assisted with data collection and analysis.

## Conflict of Interest

The authors declare that the research was conducted in the absence of any commercial or financial relationships that could be construed as a potential conflict of interest.
